# Surgical Approach and Re-tear Rates in Distal Biceps Tendon Repairs: A Single-Centre Study

**DOI:** 10.7759/cureus.98051

**Published:** 2025-11-28

**Authors:** Zain Sadozai, Samir Asmar, Dillan Mistry, Mohamed Galhoum, Mathew Varghese

**Affiliations:** 1 Trauma and Orthopaedics, Mid Yorkshire Teaching NHS Trust, Wakefield, GBR; 2 Trauma and Orthopaedics, Wrexham Maelor Hospital, Wrexham, GBR

**Keywords:** distal biceps tendon, dual incision, procedural complications, re-rupture, single incision, surgical approach

## Abstract

Introduction

Distal biceps tendon rupture is a rare yet functionally significant injury, often requiring surgical repair to restore strength and movement. Various surgical approaches exist, with the single-incision and dual-incision techniques being the most common. This study aims to evaluate whether the choice of surgical approach affects re-rupture rates in a high-volume orthopaedic centre.

Methods

A retrospective review of 259 consecutive distal biceps tendon repairs performed between January 2015 and July 2023 was conducted. All procedures were performed by sub-specialist elbow surgeons using a standardised fixation technique. Patients were categorised into single-incision (n=183) and dual-incision (n=76) groups. Re-rupture rates, post-operative complications, and functional outcomes, including Quick Disabilities of the Arm, Shoulder and Hand (QuickDASH) scores, were analysed. Statistical comparisons were performed using Fisher’s exact test and t-tests.

Results

The overall re-rupture rate was 0.38%, with only one patient experiencing a re-rupture in the single-incision group (0.55%). No significant difference in re-rupture rates was observed between the two approaches (p=1.00). At final follow-up, 90% of patients had no documented functional deficits, and 56% reported no disability based on QuickDASH scores.

Conclusion

This study demonstrates an exceptionally low re-rupture rate following distal biceps tendon repair, with no significant difference between single- and dual-incision approaches. The findings support the biomechanical integrity of both techniques. Whilst the study is limited by its retrospective design, its large sample size and standardised methodology strengthen its conclusions. Further multi-centre studies are recommended to explore additional risk factors for re-rupture.

## Introduction

Biceps tendon rupture is an uncommon traumatic injury that can be debilitating to a patient’s livelihood. With an incidence of 1.2-2.55 per 100,000 [1], it predominantly occurs in male patients and in the dominant extremity [2]. The injury itself is often a result of forced eccentric contraction of a pathological biceps tendon. Pre-existing tendon pathology has been associated with chronic inflammation or repetitive impingement of the tendon on hypertrophic bone; these are often seen in surgery. Treatment is usually operative for full rupture, and this has been shown to be the gold standard in comparison to non-operative treatment. This is primarily due to the ability to retain supination and flexion strength within the arm [3,4]. Non-operative treatment is an option; however, a 40% loss in supination strength and a 30% loss in elbow flexion strength are usually seen [1].

There are varying approaches that can be used to repair biceps tendon rupture, including the single- or double-incision technique. With the single incision being more minimally invasive, despite patient factors, surgeon choice and ability are all crucial points in determining which approach is more important [5].

Common complications are well-reported when it comes to surgery, with the most notable being lateral antebrachial cutaneous nerve injury. Within this complication field is also the possibility of re-rupture [2].

Re-rupture is perhaps not as widely seen; however, it is still a recognised complication. Re-rupture is reported to have an incidence of 1.2%-6%; however, this is very rarely reported in the literature [1]. There are many different theories for re-rupture: loss of suture or allograft purchase in the distal biceps tendon, technical failure, or suture failure [1]. Additionally, this is also the biomechanical strain that some patients can present with, resulting in overload strain on the biceps tendon.

Diagnosis of re-rupture is also a difficult clinical skill. The use of MRI can be associated with high failure rates due to difficulty establishing the biceps tendon on imaging. Despite this, persistent pain and reduced function should be associated with the suggestion to the clinician of re-rupture [1]. Our units’ approach is to identify and document clinical concern for re-rupture; this may be confirmed with imaging if there is ambiguity or to aid surgical planning.

Within our unit, we wanted to look at the failure rate of our biceps tendon repairs and, with this, try and correlate a reason for this complication. There is very little in the way of literature looking at re-rupture of biceps tendon repairs, whilst at our unit, we carry out a large volume of this procedure amongst our upper limb consultants.

The aim of our study, conducted at a single high-volume centre with a high observed incidence of distal biceps tendon injuries, is to establish re-tear rates. Our primary objective is to compare re-rupture rates between single-incision and dual-incision approaches for distal biceps tendon repairs. Our secondary objective is to assess patient satisfaction and functional outcomes with the use of a patient-reported outcome measure (PROM) following surgical intervention for distal biceps tendon repair.

## Materials and methods

This retrospective single-centre cohort study looked at consecutive distal biceps tendon repairs performed in a seven-year period between January 2015 and July 2023 in a busy orthopaedic trauma unit as part of an NHS Teaching Hospital Trust in the United Kingdom. All patients had a minimum follow-up of 12 months.

Our inclusion criteria were all patients who underwent surgical primary repair for distal biceps tendon tears between the above-stated time period. All cases performed by sub-specialist elbow surgeons at our unit were included in the study. These patients were identified by their specific operation coding using our theatre management software. Distal biceps tendon reconstruction using autograft augmentation, chronic tears, re-tear operations, and tendons found to be intact during surgery were excluded from this study.

Six upper limb surgeons perform this procedure in our unit, four of whom utilise a single-incision approach and two utilise a two-incision approach. There are small variations in technique between the surgeons of each group.

Relevant appropriate data required regarding the surgical approach, re-tear rates, and subsequent complications were collected by searching through individual patient medical records, which included their medical notes whilst in hospital, clinical letters, operation notes, and any relevant imaging. Furthermore, any patients with incomplete records were contacted by telephone to obtain further information. All patients were given a minimum of one routine clinical follow-up appointment after each of their initial surgeries. All patients had formal referral to physiotherapy as part of their post-operative instructions to rehabilitate their elbow function and monitor their range of movements. Patients were contacted at the time of the study to complete a functional outcome measure. The QuickDASH score is a subset of 11 questions from the original 30-item Disability of Shoulder and Hand score (DASH) published in 2005 [6]. It is a self-reported questionnaire and is a validated method of assessing patients with musculoskeletal disorders of the upper limb. In order for a valid score to be produced, 10 of the 11 items must be completed, and this generates a score that can range from 0 (no disability) to 100 (most severe disability).

Statistical analysis was conducted using Microsoft Excel software version 16.102.3 (Microsoft, Redmond, WA). Frequencies, means, and proportions were used to describe the data. Fisher’s exact test and t-test at the 0.05 significance level were used to compare proportions of our results.

## Results

Following screening, 259 patients who had undergone surgical repair of an acute distal biceps tendon were identified for inclusion within the study. Results are summarised in Table 1.

**Table 1:** Summary of Results

Results	Number	%
Total number of patients undergoing surgical repair	259	
Male	256	98.84
Female	3	1.16
Anabolic steroid usage	3	1.16
Single incision	183	71.48
Dual incision	76	29.69
Re-tears	1	0.38
Patients reporting loss of range of movement	21	8.20

Most patients were male (256, 98%), with an average age of 43. Only three patients within the cohort admitted to using anabolic steroids prior to sustaining a tendon rupture. Both the single- and dual-incision groups demonstrated similar demographics. No significant difference was found in average age: 42.93 (standard deviation (SD): 9.10) for the single-incision group and 43.64 (SD: 9.39) for the dual-incision group (t-test t-value=-0.57103, p=0.568476). Gender distribution between the two groups similarly showed no significant difference (Fisher’s exact test, p=0.5578).

Surgical repair of the tendon was performed by six experienced consultant upper limb surgeons within our unit. A single incision was used in 183 cases compared to 76 cases in which a two-incision approach was utilised.

One patient within the cohort developed a re-tear; this was in the single-incision group. This results in an overall incidence of 0.38% of the whole cohort and 0.55% within the single-incision group. No significant difference was noted between the single- and dual-incision groups (Fisher’s exact test, p=1.00).

A total of 238 patients had no documented loss in range of movement or weakness at their final follow-up appointments. This result demonstrates that 90% of the cohort have no documented restriction.

Successful contact at the time of study was made, and a QuickDASH score was recorded in 129 patients within the cohort. Of the respondents, 56% (72 patients) reported no disability following biceps tendon repair, with an average score of 4.9 (±2.381, 95% confidence interval).

## Discussion

Re-rupture after distal biceps tendon repair is a critical post-operative outcome, as it indicates surgical failure [7]. This patient cohort typically expects high functional and strength demands. Given the clinical significance of this issue, we conducted this study to identify potential associations with re-rupture.

In addition to the clinical significance of this topic, re-rupture following distal biceps tendon repair is rarely discussed in the literature or reported in large case studies. Due to its relatively low incidence, conducting large, single-centre studies on this complication is challenging. As a result, there is limited available data on risk factors associated with re-rupture. The primary objective of our study was to examine whether the choice of surgical approach, whilst using a consistent homogenous fixation technique, is associated with re-rupture rates. To our knowledge, this specific relationship has not been previously investigated in the literature in large case studies at a single centre.

Our results showed no difference in the re-rupture rate between the single-incision and two-incision surgical approaches. We were surprised by the low re-rupture rate observed in our study; however, this is consistent with another study of 190 patients, which reported a re-rupture rate of 1.5% [2]. This finding highlights the strong biomechanical properties of the surgical repair, as evidenced by its low failure rate. A cadaveric study evaluating failure rates following distal biceps tendon repair using an EndoButton-based technique, similar to that used in our centre, reported a failure load at 259±28 newtons [8]. Similarly, promising pullout strengths have been observed in other biomechanical analysis studies investigating alternative techniques, such as bio-tenodesis screw fixation [9].

Complications following distal biceps tendon repair have been well studied, as demonstrated by a large systematic review that analysed 3,091 primary distal biceps tendon repairs. This review found no significant effect of fixation technique on re-rupture rates [10]. Another systematic review with meta-analysis reported re-rupture rates of 2.5% in single-incision cases compared to 0.6% in two-incision cases [11]. The single-incision group included EndoButton fixation, suture anchors, and bio-tenodesis screws, whereas the two-incision group utilised only transosseous repair. However, there is limited data on the aetiology, associations, or causes of re-rupture. Most reports on re-rupture are case reports or case series. An analysis of failed repairs following cortical button fixation suggested that premature loading in the early post-operative period may contribute to failure [12]. Other studies support this hypothesis, attributing re-rupture to patient non-compliance and excessive force applied to the repair site during the immediate post-operative period [2].

The low incidence of re-rupture in our study is further supported by our patient-reported outcome measures (PROMs), which demonstrate that more than half of the cohort experienced no functional disability following surgical intervention.

The strengths of our study include the large patient population, a minimum follow-up period of 12 months, and the use of a standardised, homogenous surgical fixation technique in a single centre. However, the study is limited by its retrospective design and the potential for selection bias. Despite these limitations, this approach was necessary to achieve a sufficiently large patient cohort.

All patients had a minimum follow-up period of 12 months from surgery. The retrospective nature may have led to some patients who developed a late rupture being missed; however, as most studies identify re-rupture as a typically early complication occurring within three months, we would expect any missed case numbers to be low.

## Conclusions

In conclusion, the findings from our study, which draws strength from a large patient cohort, demonstrated no significant discernible correlation between the specific surgical approach chosen, namely, whether a single-incision or dual-incision technique was utilised, and the subsequent risk of tendon re-rupture. This study benefits from the inclusion of outcomes from several surgeons, enhancing the generalisability of our results and mitigating the risk of single-practitioner bias. This suggests that the ultimate goal of a durable repair is achievable through either primary approach, offering surgeons significant flexibility without an apparent compromise to this critical outcome. Furthermore, our study demonstrates that within our populations, repair for distal biceps tendon ruptures is a well-received intervention with patients reporting excellent outcome measure scores, indicative of successful recovery and return to function.
